# Mirror-Image Equivalence and Interhemispheric Mirror-Image Reversal

**DOI:** 10.3389/fnhum.2018.00140

**Published:** 2018-04-12

**Authors:** Michael C. Corballis

**Affiliations:** School of Psychology, University of Auckland Auckland, New Zealand

**Keywords:** bilateral symmetry, cerebral asymmetry, commissures, dyslexia, inferotemporal cortex, interhemispheric mirror-image reversal, mirror-image equivalence, symmetrization

## Abstract

Mirror-image confusions are common, especially in children and in some cases of neurological impairment. They can be a special impediment in activities such as reading and writing directional scripts, where mirror-image patterns (such as *b* and *d*) must be distinguished. Treating mirror images as equivalent, though, can also be adaptive in the natural world, which carries no systematic left-right bias and where the same object or event can appear in opposite viewpoints. Mirror-image equivalence and confusion are natural consequences of a bilaterally symmetrical brain. In the course of learning, mirror-image equivalence may be established through a process of symmetrization, achieved through homotopic interhemispheric exchange in the formation of memory circuits. Such circuits would not distinguish between mirror images. Learning to discriminate mirror-image discriminations may depend either on existing brain asymmetries, or on extensive learning overriding the symmetrization process. The balance between mirror-image equivalence and mirror-image discrimination may nevertheless be precarious, with spontaneous confusions or reversals, such as mirror writing, sometimes appearing naturally or as a manifestation of conditions like dyslexia.

## Introduction

A common source of psychological disturbance is confusion between left-right mirror-images. Although this sometimes occurs due to pathology, it is also part of the human condition and sometimes manifest in otherwise normal people under normal circumstances. Children typically go through a stage between the ages of three and seven when they confuse mirror-image letters, such as *b* and *d*, or near-mirror-image words like *was* and *saw* (Gordon, 1921). They may also confuse which way a line is sloping; Rudel and Teuber (1963) found that 3- to 5-year-old children has great difficulty choosing which of two mirror-image oblique bars (vs.) was the one designated as correct, in spite of constant feedback, but they had no difficulty with horizontal and vertical. Many of those in the 6- to 8-year range also had difficulty.

**Figure 1:**
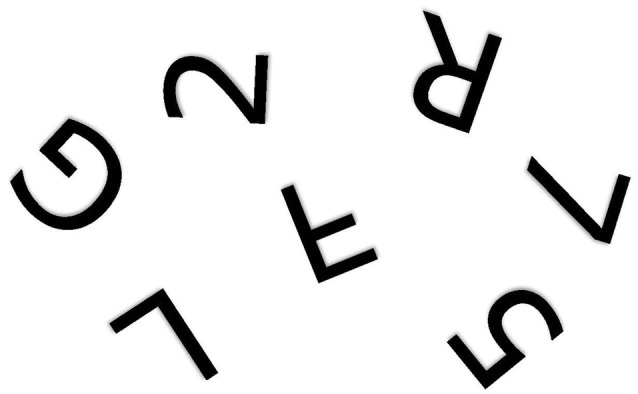
One of the alphanumeric characters shown above is a mirror image of the true character. Which one? (Author’s drawing).

The problem is fundamentally one of telling left from right (Corballis and Beale, 1970, 1976). When children first learn to write their own names from memory, they show strong tendency to write them backwards, even when using the preferred right hand (Fischer and Koch, 2016). In a letter to his friend Wilhelm Fliess, Freud (1954) once wrote: “I do not know whether it is obvious to other people which is their own or others’ right and left. In my own case in my early years I had to think which was my right; no organic feeling told me” (p. 243). Army recruits in Czarist Russia were said to have been so poor at telling left from right that they were drilled with a bundle of straw tied to one leg and a bundle of hay to the other (Elze, 1924). Discrimination of mirror-image patterns also requires reference to left and right. For example, it is not immediately apparent whether rotated letters and digits, such as those shown in Figure 1, are normal or mirror-reversed. People typically make the decision by rotating them to the upright, either physically or mentally (Cooper and Shepard, 1973; Corballis, 1988), so that the letter can then be referred to its normal left-right orientation. Failure to distinguish left from right, though, would still leave the observer perplexed. Similar arguments apply to the problem of identifying a shoe as left or right. The surest way to tell is to rotate the shoe into alignment with the feet—or simply try it on—and then label it with reference to the matching foot. But if you don’t know which foot is which, you will still be unable to label the shoe.

Of course, as adults, most of us have little difficulty, but the special problem of mirror images can be demonstrated in subtle ways. Several studies have shown people to be slower to judge whether left-right mirror images are different than to judge whether up-down mirror-images are different (Butler, 1964; Sekuler and Houlihan, 1968). Sometimes, in fact, mirror images are simply understood as the same. In one experiment people shown 2500 pictures later showed a striking ability to recognize them, except that they were as likely to report a picture as familiar if it was the left-right reverse of the original than if it was the original itself (Standing et al., 1970). The difficulty in discriminating mirror images is also widely documented in nonhuman species (Corballis and Beale, 1970, 1976). Even bees tend to treat mirror images as equivalent (Gould, 1988).

Mirror-image confusion, though, is not always an impediment, since treating mirror images as the same, or at least as equivalent, can also be adaptive. The same object can appear in mirror-image form if viewed from opposite sides, and body parts, such as hands, feet, ears and eyes, occur in mirror-image pairs. A hand is still a hand, whether left or right, although one would need to be rotated in four-dimensional space to actually match the other! Even the two sides of the brain are near-mirror images of each other. The natural world is for the most part indifferent with respect to left and right, and survival after an attack might be more likely if it is remembered as though it had impinged from either side. Objects or animals can appear in opposite, mirror-image profiles, making it useful to generalize from one to the other. For most animals there is little profit to be gained in discriminating mirror images; most of the exceptions occur in environments manufactured by humans, as in reading and writing, giving verbal descriptions involving directions, or in various conventions involved in activities such as greeting, eating or driving.

In humans, at least, there must be something of a balance between a natural tendency to treat mirror images as the same and the need to discriminate them. In what follows, I consider the neurological basis of mirror-image confusion and its converse, mirror-image equivalence (sometimes also called mirror-image generalization, or mirror-image invariance). Much of the argument rests on the fact that we humans belong to the vast clade of organisms known as the *bilateria*, which closely approximate bilateral asymmetry. This is especially true of the limbs and sense organs, and general bodily structure. People and most animals look much the same in the looking glass as they do in the real world. There are nevertheless exceptions to symmetry, notably in the internal organs and in the brain. The trade-off between mirror-image equivalence and mirror-image confusion depends to a large extent on the interplay between symmetry and asymmetry, especially in the brain.

## The Consequences of Bilateral Symmetry

I consider first the implications of bilateral symmetry. An organism that was *perfectly* bilaterally symmetrical would in fact be unable to discriminate mirror images, or tell left from right (Corballis and Beale, 1970, 1976). The simplest way to demonstrate this is to consider what would happen if everything were mirrored, as in the looking glass. A perfectly symmetrical person, or indeed any symmetrical organism or object, would be quite unaltered, but would now be seen making opposite responses to mirror images. Suppose, for example, that the person correctly responded “dee” to a *d* and “bee” to a *b*. In the mirrored world we would see the same person responding “bee” to a *d* and “dee” to a *b*. We must conclude, through *reductio ad absurdum*, that he or she would be unable to maintain consistency of response in either environment. Similarly, such a person would be unable to consistently establish which hand is the left hand and which the right. By the same reasoning a bilaterally symmetrical pigeon would be unable to consistently peck a key showing a 45-degree sloping line and refrain from pecking its mirror image—a 135-degree line. In the looking glass you would see exactly the same pigeon pecking the 135-degree line and not pecking the 45-degree one. Such a pigeon could not in fact be bilaterally symmetrical.

To put it another way, a perfectly symmetrical organism, or machine, *must* make mirror-image responses to mirror-image stimuli. It would therefore be incapable of saying “bee” to a *b* and “dee” to a *d*, or of pecking to a 135-degree line but not to a 45-degree one. Or, for that matter, of identifying one side of its body as “left” and the other as “right.”

These impediments need not prevent our symmetrical organism from correctly *seeing* the left-right orientation of an object or pattern. A symmetrical child might easily point to the round side of a *b* or *d*, yet be unable to name these letters differently. A symmetrical animal might effortlessly follow a winding trail, or approach an object to one or other side without confusion. The problem arises when labels must be applied to distinguish and object from its mirror image, or to label a turn as “left” or “right.”

**Figure 2:**
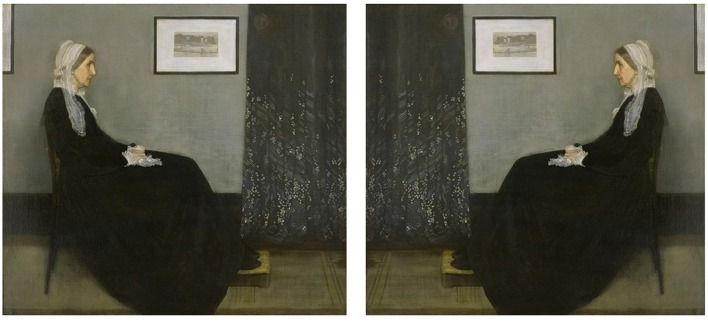
Whistler’s *Arrangement in Black and Grey No. 1*. Which version is correct? (James Abbott McNeill Whistler, Whistlers Mother high res, marked as public domain, more details on Wikimedia Commons).

Of course no human or animal is perfectly bilaterally symmetrical, but most are at least approximately so, and left-right confusions are indeed more frequent in those with lesser degrees of handedness, suggesting that bilateral symmetry does play a role (Vingerhoets and Sarrechia, 2009). But bilateral symmetry can itself be considered an adaptation to the fact that the natural world is largely indifferent to left and right—for the most part, it is adaptive to be able to reach equally well to either side, attend equally to what is happening on either side, or move in s straight line. The world in the mirror looks very much like the actual world, and again the exceptions arise mainly in the world constructed by humans. This may well explain why children, in particular, have difficulty learning to read scripts that are laid out in a consistent direction, and why even adults may have difficulty remembering which way round things are. For example, if your coins show the profile of a sovereign or notable person, can you say which way round it is? Figure 2 shows two versions of Whistler’s famous portrait of his mother, *Arrangement in Black and Grey No. 1*. Which one is correct?

The difficulty in discriminating mirror images seems to persist even through asymmetrical experience, although most of us eventually overcome it. In some children, especially those classified as dyslexic (discussed later), it persists in mirror-writing or in wrongly labeling letters such as *b* and *d* even though the experience of looking at script in books or the teacher’s copy is overwhelmingly asymmetrical. This suggests that the brain has a natural tendency to preserve symmetry in the face of asymmetrical experience, and so to treat mirror images as equivalent.

## The Role of the Temporal Cortex

One area of the brain involved in mirror-image equivalence is the inferotemporal cortex. Single cells recorded in the inferotemporal cortex in infant monkeys responded to both a face and its mirror image, indicating mirror-image equivalence early in development (Rodman et al., 1993). The monkey’s face-processing system also seems to be organized hierarchically, with one patch responding to faces from particular viewpoints, another patch responding to a given viewpoint and its mirror image, and another responding to almost any viewpoint (Freiwald and Tsao, 2010). Mirror-image equivalence, then, may be a way station toward full shape invariance.

Logothetis et al. (1995) found that some single cells in the inferotemporal cortex of two adult rhesus monkeys responded equivalently to meaningless mirror-image shapes, and remarked that “Distinguishing mirror images has no apparent usefulness to any animal” (p. 360). More recently, Rollenhagen and Olson (2000) recorded from pattern-selective cells in the inferotemporal cortex of the macaque, and again some cells responded more similarly to left-right mirror images than to up-down mirror images, even when the stimuli appeared at different locations across the visual field. They suggest that this mirror-image equivalence “is a direct correlate of lateral mirror-image confusion as observed in perception” (p. 1508)—although the confusion is almost certainly a matter of recognition rather than perception *per se*. Baylis and Driver (2001) showed similarly that shape-coding cells in the inferotemporal cortex of the monkey generalize between mirror images and patterns with reversed contrast, but not between figure-ground reversals.

The fusiform gyrus, which extends from the occipital into the inferior temporal lobe, seems to be host to representations of different categories of visual input, such as faces, objects, words, and scenes. In the chimpanzee, both sides are involved in face recognition. In humans, the right fusiform includes the fusiform face area (FFA), while the corresponding region in the left is specialized for the processing of written words, and is known as the visual word form area (VWFA). This difference is evident in structure as well as in function; in humans, the neural minicolumns in the fusiform gyrus are wider on the left than on the right, an asymmetry not apparent in the chimpanzee (Chance et al., 2013). Developmental studies show that the asymmetry is not evident in children before they learn to read, but the left fusiform asymmetry then emerges as they gain proficiency. The right-fusiform advantage for face recognition of faces appears to be further delayed until adulthood (Behrmann and Plaut, 2015). In humans, the emergence of literacy appears to have commandeered the left side of the fusiform gyrus for word recognition, thus creating the asymmetry necessary for mirror-image discrimination. Dehaene and Cohen (2011) describe this process as the “recycling” of cortical territory, originally designated for object and face recognition, for the recognition of written words.

A critical feature of word-recognition is that it requires left-right discrimination, whereas recognition of other natural patterns does not. Using fMRI, Dehaene et al. (2010a) showed that activity in the VWFA is suppressed when identical pairs of words or pictures are presented to adult readers, indicating a priming effect. In the case of pictures, presenting mirror-image pairs also induced suppression in a region of the occipitotemporal cortex close to the VWFA, but with similar profiles on the left and right. These areas apparently underlie mirror-image equivalence for pictures. In the case of words, though, VWFA suppression was essentially absent, even though the area concerned overlapped with area showing the mirror-image equivalent for pictures. Dehaene et al. (2010a) conclude that “learning to read recruits an area which possesses a property of mirror invariance, seemingly present in all primates, which is deleterious for letter recognition and may explain children’s transient mirror errors” (p. 1837).

Two other studies, though, do show evidence of mirror-image priming for words when participants are engaged in the more effortful task of reading mirrored words. For both normal and mirrored primes, activity was suppressed in areas that included an area very close to the VWFA (Lin and Ryan, 2007; Ryan and Schnyer, 2007). This suggests that the VWFA can recruit resources for processing mirror-reversed words when the task demands it. In another priming study in adults, Borst et al. (2015) showed that there was a cost involved in blocking the tendency to treat mirror-image letters as equivalent, suggesting that “expert readers never completely “unlearn” the mirror-generalization process and still need to inhibit this heuristic to overcome mirror errors” (p. 228).

The responses in the fusiform gyrus can be contrasted with responses earlier in visual processing, where left-right orientation is maintained. In a brain-imaging study, Dilks et al. (2011) found that when people were shown pairs of pictures of objects in sequence the object-selective region responded equally whether the two object were the same or mirror images, but less when they were two different objects. Recordings from an earlier stage in the processing stream, the lateral occipital sulcus, showed a lesser response when the objects were mirror images, suggesting that mirror-image discrimination was registered at this stage but lost later. Axelrod and Yovel (2012) also report mirror-image equivalence for faces in the fusiform gyrus as well as in the superior temporal sulcus, but sensitivity to left-right orientation in the earlier occipital face area. Thus, early processing retains left-right information for perception, but this is lost at the later stage where recognition takes place.

The asymmetry of the VWFA may be partly a product of literacy itself, leading not only to the breaking of symmetry but also to the discrimination of words and letters from their mirror images. Dehaene et al. (2010b), using fMRI, compared brain responses in literate and illiterate people, and found that literacy not only resulted in the responsiveness of the left-hemispheric VWFA to script, but also enhanced early responses in the visual cortex. It also allowed the entire left-hemispheric language circuit to be activated by written sentences, and even enhanced phonological responses in the left temporal planum, one of the prominent speech areas. Literacy is essentially a late cultural phenomenon and is still far from universal (although rapidly increasing), but it appears to command the language system as efficiently as speech itself, which is generally considered universal and a consequence of biological evolution. But literacy may have negative consequences as well. It results in reduced activation in the VWFA to faces and checkerboards, leading Dehaene et al. (2010b) to speculate that perception of faces may suffer as literacy develops.

## Symmetrization

Given that left-right equivalence is a natural consequence of bilateral symmetry, and that left-right equivalence applies even to perceptual and motor learning, we may postulate an active process of symmetrization in the formation of memory circuits. The simplest mechanism for achieving this would be through interhemispheric transfer in the course of memory formation. If the commissures connect mirror-image points in the two hemispheres, activation in the transmitting hemisphere would be mirrored in the receiving hemisphere, which would therefore record the information as though left-right reversed. This process has been called *interhemispheric mirror-image reversal* (Corballis and Beale, 1970). Thus each hemisphere correctly perceives the symbol *b*, which is then registered in storage, but in the storage process the information is also transferred and reversed between hemispheres. This means that the brain as a whole stores both *b* and *d*, achieving both bilateral representation and mirror-image equivalence.

**Figure 3:**
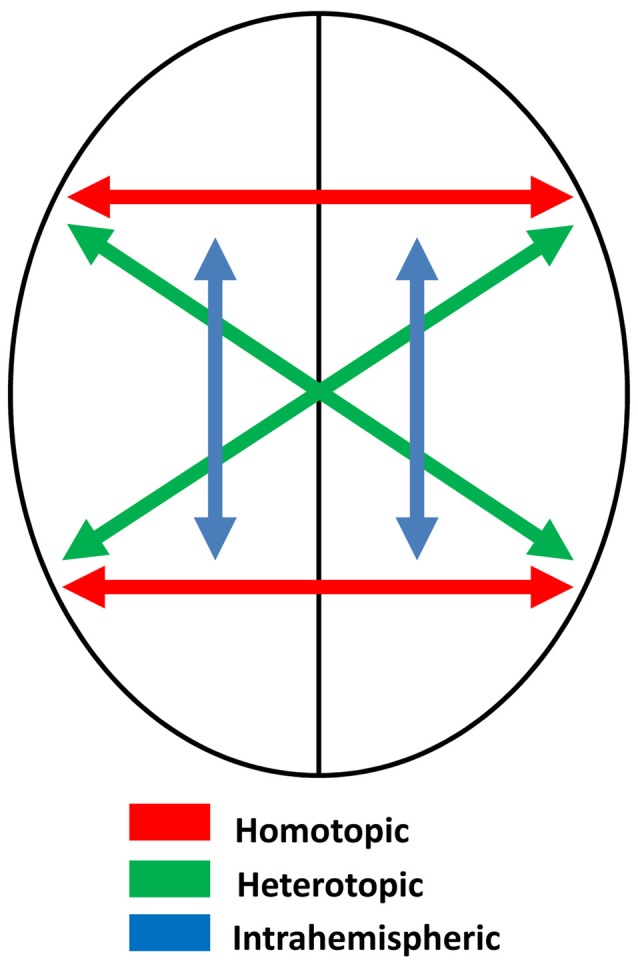
Schematic diagram illustrating different kinds of neural connections in the brain: homotopic, heterotopic and intrahemispheric.

Assuming that the two sides of the brain are themselves mirror images, and that connections between them are homotopic—that is, they connect mirror-image point—the process of memory transfer should maintain symmetry and create memory circuits that preserve mirror-image equivalence. Some of these connections, though, are heterotopic, not homotopic (Clarke, 2003; Chovsepian et al., 2017)—see Figure 3. These include connections between the visual cortices (e.g., Zeki, 1971), which probably serve to maintain perceptual continuity across the midline. Single cells in the occipital cortex of the macaque have receptive fields that are primarily contralateral, but with increasing ipsilateral representation as recording moves from primary visual cortex to extrastriate areas (Van Essen et al., 1982), and brain imaging in humans also shows increasing ipsilateral representation in the progressions through visual to parietal areas (Jack et al., 2007). Ipsilateral representation depends on interhemispheric transfer via the corpus callosum, and this transfer must be heterotopic rather than homotopic, preserving orientation as perceived objects cross from one visual field to the other (Berlucchi, 2014). Thus the letter *b*, for example, is *perceived* correctly regardless of which visual field it appears in.

Most of the fibers in the corpus callosum, though, are homotopic (de Lacoste et al., 1985; Clarke and Zaidel, 1994; Hofer and Frahm, 2006; Roland et al., 2017), and it may be these fibers, or some of them, that establish memory circuits on one side of the brain mirroring those on the other. In primates, the anterior commissure also has both heterotopic and homotopic fibers, and connects frontal and temporal areas, with especially dense connections between the inferotemporal cortices (Di Virgilio et al., 1999; Schmahmann and Pandya, 2006), which as we have seen are known to be involved in visual learning. As I suggest below, the anterior commissure may be especially involved in mirror-image equivalence. Such a mechanism would apply regardless of the nature of the storage, and does not require that representation be somehow picture-like or analog. So long as the mechanism operates to preserve bilateral symmetry, the brain would fail to record mirror-images as distinct. Further, it need not be the case that memories are installed separately in the hemispheres. It is conceivable that some memory circuits straddle the hemispheres, but these too would be “symmetrized” in the course of homotopic transfer.

Early evidence for interhemispheric mirror-image reversal came from research with pigeons. Since each eye projects wholly to the contralateral side of the brain, interhemispheric transfer of memory can be tested by teaching a discrimination through one eye, and then testing with the other eye. Mello (1965) taught pigeons monocularly to peck a key displaying an oblique line, and tested responses to lines of varying orientation with each eye in turn. When tested with the trained eye, responses peaked at the trained orientation, but with the untrained eye the responses peaked at the mirror-image orientation. This suggests that the learning itself was mirror-reversed in the untrained hemisphere. She reported similar reversals with other mirror-image patterns (Mello, 1966). These studies were not entirely unequivocal, because the birds might well have been coding the stimuli in terms of relation to the beak. This would explain reversal since the stimulus is on opposite sides of the beak when viewed with opposite eyes, and the commissures themselves may have played little role (Beale and Corballis, 1968).

A further study, though, suggested that the commissures do play a role. When trained with both eyes to peck a key displaying an oblique line, pigeons tested on varying orientations of the line show peaks at both the trained orientation and the untrained one, demonstrating mirror-image generalization. When the commissures were sectioned, however, the peak at the mirror-image line was abolished (Beale et al., 1972). This can be taken to mean that mirror-image generalization normally depends on the commissures.

Noble (1966) reported a similar reversal in monkeys with section of the optic chiasm, so that visual input to one eye was projected only to the contralateral hemisphere and interhemispheric transfer of learning then tested through the other eye. Other studies, though, have suggested a lack of transfer rather than reversal in both cats (Berlucchi and Marzi, 1970) and monkeys (Noble’s, 1968; Hamilton and Tieman, 1973), although transfer of discriminations was stronger when nonmirror-image discriminations were tested. This suggests that there may be conflict between the reversed trace and the nonreversed one. That is, input to the untrained hemisphere may either be compared to the reversed memory circuit in that hemisphere, or transferred veridically to the trained hemisphere for comparison with the nonreversed circuit.

### A Role for the Anterior Commissure?

Noble’s (1968) experiment provided further information undetected by Noble himself. He trained and tested monkeys on both left-right and up-down discriminations. On some tests the corpus callosum was sectioned, on some the optic chiasm was sectioned, and on some both were sectioned. In some cases where the corpus callosum was sectioned, the anterior commissure was spared, and in other cases it was not. A reanalysis of the data showed that the monkeys were much poorer at learning to discriminate left-right mirror images than up down ones, *except when the anterior commissure was sectioned* (Achim and Corballis, 1977). This raises the possibility that interhemispheric reversal, and mirror-image equivalence, may depend at least partly on the anterior commissure rather than on the much larger corpus callosum.

This must be true of the pigeon, which has an anterior commissure but no corpus callosum, a phylogenetically more recent structure exclusive to placental mammals (Mihrshahi, 2006; Suárez et al., 2014). In primates the anterior commissure has widespread cortico-cortical connections in the frontal, temporal and parietal areas (Wei et al., 2017), particularly in the temporal and frontal lobes, with especially dense projections in the inferior part of the temporal lobe (Di Virgilio et al., 1999). It may play little role in the transfer of perceptual information, at least in humans, since section of the corpus callosum alone results in perceptual disconnection apparently identical to that following section of both corpus callosum and anterior commissure (Gazzaniga, 2005). This raises the possibility that the anterior commissure may be specialized for memory transfer, making little contribution to perceptual transfer.

### Hippocampal Commissure

The hippocampal commissure has been somewhat neglected in studies of interhemispheric transfer, but is nonetheless a further candidate for symmetrization of memory circuits, given the critical role played by the hippocampus itself in memory formation. Evidence for transfer comes mainly from observations of seizures, which can sometimes spread from one hemisphere to the other. For instance *in vitro* studies in rats show that seizures induced in the hippocampus on one side can lead to a secondary mirror-focus on the other side, implying long-term synaptic change carried by the hippocampal commissure (Khalilov et al., 2003). In rats, too, bilateral seizure activity is tightly synchronized, with very short delays (<2 ms), implying transmission over the short distance of the hippocampal commissure rather than a more circuitous route (Wang et al., 2014).

In rats the hippocampal commissure is dominated by the ventral portion, which is reduced in primates and vestigial or possibly absent in humans. The dorsal hippocampal commissure, though, is clearly present in humans, and is a sizeable tract (Lacuey et al., 2015). Gloor et al. (1993) proposed that delayed transfer of amnestic seizure from the mesial temporal lobe in one hemisphere to the contralateral region of the other was carried by the dorsal hippocampal commissure. More recently, using single-photon emission computerized tomography (SPECT), Huberfeld et al. (2006) found that in 57.1 percent of cases with temporal-lobe epilepsy a mirror focus was established in the other hemisphere. They too suggest that the dorsal hippocampal commissure may be involved, perhaps along with the anterior commissure and corpus callosum. Lacuey et al. (2015) found that deep stimulation to the fornix on one side induced a response in the contralateral side in a few (but not all) patients with focal epilepsy without involvement of the temporal cortex or amygdala, again suggesting transfer via the dorsal hippocampal commissure.

In summary, interhemispheric reversal may well underlie mirror-image equivalence, and by the same token mirror-image confusion as well, through the establishment of memory circuits that correspond both to a learned event and to its mirror image. With respect to visual patterns, the inferotemporal cortex in primates may be critical, and the reversal may be dependent on homotopic transfer of learning. The anterior and hippocampal commissures may be more critical to this process than the corpus callosum. One way to test this conjecture would be to section these commissures in nonhuman primates and test for mirror-image discrimination. The expectation is that mirror-image discrimination would be accomplished more readily following commissural section.

### Motoric Asymmetry

The ability to tell left from right may depend also on motor asymmetries. Most people have a dominant hand, usually the right, and this provides a consistent asymmetry that can offset the mirror-image problem. Confusions between mirror-image letters or words can be resolved with reference to the hand used to write them, or the direction in which they are written. Even so, the non dominant hand may intrude with a mirroring influence. Indeed, the limbs are innately programmed to operate in mirrored fashion. Walking, running, swimming and flying all involve mirrored movements, whether in succession or simultaneously.

Even skills learned with one hand may be reversed with the other, providing further evidence for interhemispheric mirror-image reversal. In one early study, people were taught to move a stylus around a clover-leaf slot, using their right hands. Later tested with the left hand, they proved faster at moving the stylus in the opposite direction (Milisen and Van Riper, 1939). Luria (1970) wrote of a man who, following injury to his right parietal lobe, drew a left-right reversed map of Russia, although the verbal labels on the map were written normally. A more common example is mirror-writing, which is sometimes produced spontaneously in the absence of pathology, or sometimes when the dominant arm is temporarily incapacitated (Schott, 1980). Almost all normal 5-year-olds write backwards at some stage, and this depends more on context, such as where the pen is on the page, than on which hand is used or whether the child is male or female (Fischer and Tazouti, 2012).

Mirror-writing can also occur naturally. If asked to write simultaneously with both hands, most people write forwards with the dominant hand but backwards with the nondominant one. It is surprisingly easy to write in mirror fashion even with the dominant hand, as when writing underneath a board or one one’s forehead (Critchley, 1928), where the spatial sense dominates over the motor habit. Spontaneous mirror writing is more common in left-handers or mixed-handers than in right-handers (Ireland, 1882; Schott, 2007). This is consistent with evidence that left- and mixed handedness results from the lack of a genetically induced bias toward right-handedness (Annett, 2002; McManus, 2002; McManus et al., 2013), so that habits may be as readily controlled by one hemisphere as the other. The classic case is Leonardo da Vinci, a left-hander who wrote in mirrored script in his notebooks but normally in correspondence.

The idea that mirror-writing might be due to interhemispheric mirror-image reversal, with the skill established in one hemisphere reversed in transfer to the other, was proposed by Brain (1965):

The fact that learning to write includes an unconscious education in mirror-writing, especially with the left hand, implies the establishment, probably in the right hemisphere, of graphic motor-schemas which are the mirror-images of those which underlie normal writing in the left hemisphere (p. 134).

It is much more frequent following left-hemisphere than right-hemisphere damage, and much more frequent in writing with the left than with the right hand (Balfour et al., 2007; Schott, 2007), supporting the view that the left-handed writing is controlled by a reversed memory circuit in the right hemisphere. Schott also points out, though, that left-sided lesions resulting in mirror-writing are extremely variable, including the basal ganglia, striatum and internal capsule, thalamus, and areas in temporal, parietal and frontal cortices and their overlaps. This variability, he suggests, make it unlikely that any specific focal area in the left-hemisphere is responsible for mirror-writing. Even so, left-sided damage in different regions may tip the balance toward intact but reversed circuits in the right hemisphere.

**Figure 4:**
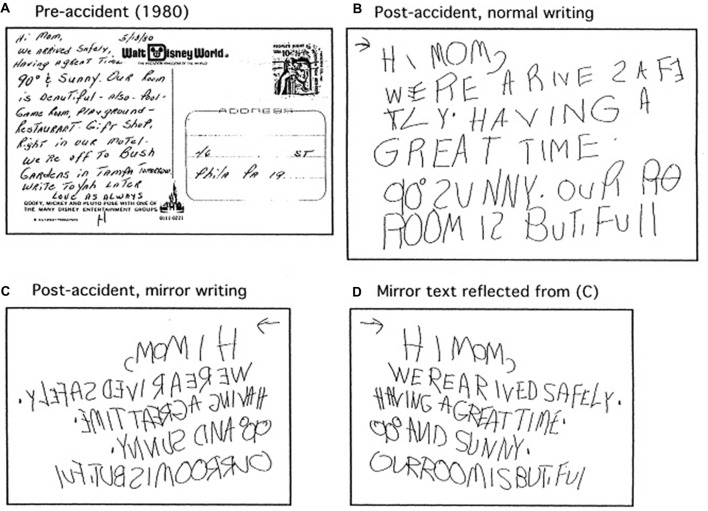
Examples of normal and mirrored writing by a woman following a cardiovascular accident (from Gottfried et al., 2003). **(A)** Writing before the accident. **(B)** Normal writing after the accident. **(C)** Mirror writing after the accident. **(D)** Mirror writing after the accident shown left-right reflected. Permission to reproduce by agreement between MCC and Elsevier, license number 4275571496658.

Sometimes, reading is also mirrored, although it is much less commonly reported than mirror-writing. In one report, a 51-year-old right-handed woman suffered brain injury following a motor vehicle accident, and thereafter preferred to write backwards with either hand, and could read mirrored words more rapidly than normal words (Gottfried et al., 2003). Examples are shown in Figure 4. The reversal was evident only in reading and writing. She showed no such reversal or left-right confusion with pictures, spoke normally, and had no problems with movements or gait. Before the accident she read and wrote normally. Brain imaging showed no obvious brain injury, but one must suspect subtle injury that somehow suppressed the normal habits in reading and writing, and released the reversed ones.

Another case, an intelligent Australian woman and member of Mensa, suffered a cardiovascular accident of the left hemisphere with right hemiplegia, and thereafter wrote much more easily backwards with the left hand, and read words and letters much more rapidly when presented mirror-reversed than when presented normally. But she showed reversals in activities other than reading and writing, such as numbering the plates on a baseball field in reverse (clockwise) order. She correctly drew a clock face but read clock times as though the clock were reversed, so that 3:00 pm was read as 11:45. Given the task of drawing countries, she drew five of six countries left-right reversed, but drew the sixth normally (Lambon-Ralph et al., 1997). That country was New Zealand, perhaps looked down upon by Australians, altering their perspective. Left right confusions are often idiosyncratic.

## Dyslexia—A Paradigm Case

In work carried out in the 1920s and 1930s, the American physician Orton (1937) proposed that dyslexia was a consequence of left-right confusions and reversals, due to poorly established cerebral dominance, which he linked to left- or mixed-handedness. Orton argued that mirror-image memories were formed simply because the two sides of the brain were themselves mirror images, and therefore must record images in mirror-image fashion (Orton, 1937). That is, one side of the brain must store memories as though mirror-reversed, preserving mirror-image equivalence in the brain as a whole. Thus a child might store the symbol *b* correctly in one side of the brain, but as though it were a *d* in the other. Mirror-image confusion would occur because of failure to suppress the reversed memory, which in turn could arise through failure to develop cerebral dominance. Orton dubbed this condition *strephosymbolia* (“twisted symbols”).

Orton’s theory cannot be correct as it stands, because there is no reason to suppose a hemisphere would directly record events or objects as they impinge on the senses as though mirror-reversed. It makes little sense for one side of the brain to actually perceive the world as though in a mirror, even if that side is the mirror image of the other. The more likely possibility is that both sides of the brain perceive the world normally, but that mirror-image storage is achieved through the process of interhemispheric mirror-image reversal, as outlined above. In this way, the brain would tend to retain structural symmetry in the face of asymmetrical experience, and so maintain mirror-image equivalence.

Orton may well have been correct, though, in linking at least some forms of dyslexia with poorly established cerebral dominance. A case in point is the American author Eileen Simpson, who suffered from dyslexia as a child, and in her book *Reversals* wrote of her persistent tendency to read the word “was” as “saw” causing her exasperated aunt to exclaim “No. How can you be so stupid? The word is ‘was’ WASWASWAS” (Simpson, 1980). Simpson also described herself as a natural left-hander who had been forced to write with the right hand, perhaps leading to poorly established cerebral dominance one way or the other.

Evidence on the relation between dyslexia and handedness, though, is mixed. Geschwind and Behan (1982), based on a large sample, proclaimed that dyslexia was 11 times more frequent in strong left handers than in strong right handers, although a review of studies by Bishop (1990) found little support for an association with handedness. More recent work (e.g., Vlachos et al., 2013) suggests that non-right-handers are indeed more prone to dyslexia than are right-handers, but the association is weak. Handedness is in any case a poor proxy for cerebral asymmetry; it is only weakly related to cerebral asymmetry for language and not related at all to asymmetry for spatial attention (Badzakova-Trajkov et al., 2010).

As Orton had observed, mirror-image confusions and reversals do seem to linger longer in children diagnosed as dyslexic than in children who learn to read normally (Fernandes and Leite, 2017). Lachmann and van Leeuwen (2007) write that “children with dyslexia fail to suppress symmetry generalization” (p. 73). By fifth-grade, dyslexics seem to have a special difficulty, not just with mirror-image letters but also with meaningless mirror-image shapes. Moreover, they are actually better than normal readers at seeing that mirror images have the same basic shape—thus a *b* can be seen as the same as a *d*, rotated about the vertical, or viewed from the other side. The problem with mirror images, moreover, is highly specific; dyslexic children have no problem with shapes rotated in the picture plane, in which *d* and *p* are the same (Fernandes and Leite, 2017).

Two meta-analysis of brain activity during reading or reading-related show underactivation in left temporal and parietal areas in people classified as dyslexic compared to normal readers (Maisog et al., 2008; Richlan et al., 2009). This pattern was confirmed in later meta-analysis that also showed that underactivation was more pronounced in a language with deep orthography (English, with inconsistent mapping between graphemes and phonemes) than in those with shallow orthographies (Dutch, German, Italian, Finnish and Swedish, where the mapping is more regular; Martin et al., 2016). People with dyslexia also show anomalies of brain structure. Frye et al. (2010) found that the surface are of the left fusiform gyrus was larger in dyslexic adults than in normal readers, which can be interpreted to imply increased gyrification and weaker connectivity (Van Essen, 1997). A meta-analysis based on fractional anisotropy (FA) also shows weaker intrahemispheric connectivity in dyslexic compared to typical readers, especially in the temporoparietal region (Vandermosten et al., 2012).

In contrast, FA analysis suggests higher connectivity in the corpus callosum in dyslexic readers than in normal readers (Frye et al., 2008). Vandermosten et al. (2012) suggest that “dyslexic readers [might] have a more infantile language network, namely a better connectivity (i.e., higher FA) in the posterior part of the CC and a lower FA in association tracts” (p. 1547). This might be taken as consistent with the hypothesis that interhemispheric reversal underlies mirror-image equivalence, while mirror-image discrimination depends on the elaboration of lateralized circuits, and might account for the finding that dyslexic readers show enhance equivalence is enhanced mirror-image equivalence but deficient mirror-image discrimination (Lachmann and van Leeuwen, 2007).

Such anomalies may have a genetic basis. Skeide et al. (2016) found that a candidate gene for dyslexia, NRSN1, was associated with volume of the VWFA, and that NRSN1-associated volume determined before school could predict later dyslexia with over 70 percent accuracy. Although the formation of the VWFA may be partly a product of literacy itself, as suggested above, it appears also to be genetically constrained.

Although these various findings provide broad support for the association between mirror-image confusions and anomalies of cerebral asymmetry, Orton’s theory and the variant suggested above have largely lost favor. Current theories emphasize a failure of grapheme-to-phoneme mapping rather than left-right problems (Peterson and Pennington, 2015). The two views are not necessarily incompatible; dyslexic individuals may have difficulty in mapping letters to sounds because they have difficulty discriminating the letters in the first place. Even so, some dyslexics seem to have difficulty discriminating phonemes independently of reading (e.g., Power et al., 2016), although it is possible that literacy itself may sharpen phonemic awareness.

Poorly established cerebral dominance may be related to language generally, rather than being specific to reading. Bishop et al. (2014) found that 4-year-old children with language impairment, unlike those with normal language development, showed no left-hemispheric bias in language processing. Curiously, though, the converse does not seem to hold; children who lack cerebral dominance do not appear to suffer any cognitive deficits (e.g., Knecht et al., 2001). This suggests some other factor, perhaps genetic, that underlies both language impairments and poorly developed cerebral dominance. Another possibility, suggested by Bishop (2013), is that weak cerebral asymmetry may be an outcome of weak language learning. The relations between language generally, reading, cerebral asymmetry and interhemispheric connectivity require further disentangling.

## Conclusion

Among the bilateria, the body and brain have evolved to be largely bilaterally symmetrical, an adaptation to the indifference of the natural world to left and right. The pressure toward symmetry appears to be maintained in ontogeny as well as phylogeny; that is, not only are the body and brain constructed on a bilaterally symmetrical plan, but we are also equipped with mechanisms to maintain at least a degree of structural symmetry in the brain despite asymmetrical experience. Through a process of symmetrization, we tend to remember and learn things that are not only as experienced, but also as the left-right reverse of the experience. This is why children often have difficulty learning to read directional scripts, and why even some adults may have occasionally have difficulty remembering which is left and which is right. The problem persists in some individuals with dyslexia.

The main thesis of this article is that symmetrization is achieved through a process of interhemispheric mirror-image reversal during the establishment of memory circuits. This is perhaps the simplest mechanism to explain why people and animals tend to confuse remembered patterns with their lateral mirror images, even when there has been little or no experience with the mirror images themselves. It is sufficient that the process simply acts to restore bilateral symmetry in the brain, since mirror-image equivalence is a necessary consequence of bilateral symmetry. This theory also explains why mirror-imaging sometimes arises spontaneously, since mirrored circuits can be maintained, albeit suppressed, along with the veridical ones, and can intrude if the balance is disturbed.

Of course, humans do learn to discriminate mirror images, albeit with some persisting confusion. Because both mirror-image equivalence and mirror-image discrimination are adaptive in different contexts, the balance between the two is sometimes precarious. Discrimination itself may depend on a weakening of the transfer process, so that reversed circuit is established more weakly, or it may depend on the dominance of one hemisphere to record a memory in the first place. Cerebral dominance for language and for manual activity may well be under at least partial genetic control, and underwrite the shift from mirror-image equivalence to mirror-image discrimination, especially in those very domains where they are of importance. As we have seen, the circuits underlying literacy seem to ride on those underlying language itself, which provides the asymmetry necessary to learn the directional aspects of reading. Handedness too appears to be under at least partial genetic control (Medland et al., 2009), and may be detected as early as the first trimester of the human fetus (Hepper et al., 1998).

Genetic control appears to vary, though, between a unidirectional shift, rightward in the case of handedness and leftward in the case of cerebral dominance for language, and the absence of a directional shift so that the asymmetries are a matter of chance (Corballis et al., 2012; McManus et al., 2013). This means that left- and mixed-handers may be especially vulnerable to left-right confusions, but better equipped for aspects of visual perception and manual action. The artistic and mechanical genius of Leonardo da Vinci may be a case in point. Variability itself may be adaptive, and maintained perhaps by a heterozygotic advantage, ensuring an allelic mix (Annett, 1985; Corballis, 1997). But we all, right- and left-handers alike, maintain a strong tendency toward mirror-image equivalence, especially in activities that are better served by symmetry, or by maintaining no distinction between mirror images. But this tendency must be over-ridden in activities where it is important to remember mirror images as distinct. This is especially the case in activities that are peculiarly human, such as reading and writing, and the maintenance of conventions that favor one or other hand. Sometimes, though, asymmetry may have more general advantages, as perhaps in complex mental operations like language that are better served by circuits unconstrained by symmetry.

The conflict between symmetry and asymmetry plays out not only in the brain but in its external manifestations, such as art and architecture. Madame de Maintenon, second wife of Louis IV of France, wrote of her husband that “he thinks of nothing but grandeur, magnificence, and symmetry.” But symmetry meant that windows and doors in the palace were placed opposite one another, creating draughts, so she went on, famously, to write “you must perish in symmetry” (quoted in Anon, 1855, p. 428). The mathematician Weyl (1950), in his treatise on *Symmetry*, reported that most people judge symmetrical shapes to be more pleasing than asymmetrical ones, but artists and creative people prefer asymmetry. Many have supposed that human variation depends on the relative contributions of the left and right hemispheres, but the more telling axis may be that between symmetry and asymmetry.

## Author Contributions

The author confirms being the sole contributor of this work and approved it for publication.

## Conflict of Interest Statement

The author declares that the research was conducted in the absence of any commercial or financial relationships that could be construed as a potential conflict of interest.
